# Decreased Risk of Stroke in People Using Red Yeast Rice Prescriptions (LipoCol Forte®): a Total Population-Based Retrospective Cohort Study

**DOI:** 10.1155/2022/8160425

**Published:** 2022-04-23

**Authors:** Chuen-Chau Chang, Mao-Feng Sun, Yi-Chun Chou, Chun-Chieh Yeh, Chaur-Jong Hu, Yih-Giun Cherng, Ta-Liang Chen, Chien-Chang Liao

**Affiliations:** ^1^Department of Anesthesiology, Taipei Medical University Hospital, Taipei, Taiwan; ^2^Anesthesiology and Health Policy Research Center, Taipei Medical University Hospital, Taipei, Taiwan; ^3^Department of Anesthesiology, School of Medicine, College of Medicine, Taipei Medical University, Taipei, Taiwan; ^4^School of Chinese Medicine, College of Chinese Medicine, China Medical University, Taichung, Taiwan; ^5^Department of Physical Medicine and Rehabilitation, China Medical University Hospital, Taichung, Taiwan; ^6^Department of Surgery, China Medical University Hospital, Taichung, Taiwan; ^7^Department of Surgery, University of Illinois, Chicago, IL, USA; ^8^Division of Neurology, Department of Internal Medicine, Shuang Ho Hospital, Taipei Medical University, Taipei, Taiwan; ^9^Department of Anesthesiology, Shuang Ho Hospital, Taipei Medical University, Taipei, Taiwan; ^10^Department of Anesthesiology, Wan Fang Hospital, Taipei Medical University, Taipei, Taiwan; ^11^Research Center of Big Data and Meta-Analysis, Wan Fang Hospital, Taipei Medical University, Taipei, Taiwan

## Abstract

The influence of red yeast rice (RYR) on the risk of incident stroke remains underexplored. We aimed to compare the risk of stroke between people with and without use of RYR prescriptions. We used research data from the National Health Insurance Program in Taiwan and identified 34,723 adults (aged ≥20 years) who first received the RYR prescription from 2010 to 2014. To select the appropriate control group, we used frequency matching by age and sex (case-control ratio = 1 : 1) and identified a non-RYR cohort that included 34,723 adults who first received lovastatin. Events of an incident stroke that occurred during the follow-up period of 2010–2017 were identified from medical claims. The adjusted hazard ratios (HRs) and 95% confidence intervals (CIs) of stroke risk associated with RYR prescription were calculated in the multiple Cox proportional hazard model. Compared with the non-RYR cohort, patients who received RYR prescriptions had a decreased risk of stroke (HR 0.65, 95% CI 0.59–0.71), including hemorrhagic stroke (HR 0.60, 95% CI 0.44–0.83), ischemic stroke (HR 0.49, 95% CI 0.43–0.57), and other types of strokes (HR 0.53, 95% CI 0.42–0.67). The association between RYR prescription and stroke risk was significant in both sexes and in people aged more than 40 years, as well as in those individuals with various medical conditions. The frequency of RYR prescription (HR 0.57, 95% CI 0.50–0.64) was associated with a decreased risk of stroke with a dose-response relationship (*p* for trend<0.0001). This study showed a potentially positive effect of RYR on the risk of stroke. However, compliance with medication use should be cautioned. The findings of this study require future studies to validate the beneficial effects of RYR prescription on stroke risk.

## 1. Introduction

Stroke remains as one of the leading causes of death and disability due to the fact that it was estimated that nearly 13.7 million people experienced new strokes and that 5.5 million people died from stroke in 2016 throughout the world [1–4]. Almost 5% of all disability-adjusted life years and 10% of all deaths throughout the world are due to stroke [2]. According to statistics from the American Heart Association, the economic costs of stroke treatment and poststroke care can substantially impact family and society burdens [4]. The epidemiology, prevention, and treatment of stroke have been well established, and hypercholesterol is known for being a major risk factor for stroke. Although statins have been proven to effectively control the levels of total cholesterol and are also widely used throughout the world, the risk of diabetes after treatment with statins has also attracted attention [5, 6].

Red yeast rice (RYR), which is also known as *Monascus purpureus* Went rice, is an herbal medicine that has been frequently used among patients with hypercholesterol in the Chinese population, and its therapeutic effects have been previously investigated [7–10]. Scientifically processed RYR prescriptions (such as Xuezhikang®, HypoCol®, and LipoCol Forte®) that contain monacolin K (lovastatin) have been proven to effectively reduce the levels of total cholesterol and low-density lipoprotein cholesterol [11–13]. In addition, a recent study found that people who used RYR had a lower risk of diabetes than people who used statins [14]. However, more assessments of the potential side effects of RYR are needed, although the tolerance and safety of the RYR use has been previously reviewed and studied [9, 15, 16].

A systematic review and meta-analysis evaluated seven clinical trials and suggested that RYR was associated with improved cardiovascular outcomes and lipid profiles in myocardial infarction patients with borderline hypercholesterolemia [17]. Nevertheless, limited information is available on the comparison of the risk of incident stroke between people who did and did not use RYR. By using reimbursement claim data from the National Health Insurance Program in Taiwan, we conducted a retrospective cohort study with a real-world database to evaluate the effects of RYR prescriptions on the risk of incident stroke.

## 2. Methods

### 2.1. Source of Data

We conducted this study by using research data from the National Health Insurance in Taiwan. This insurance program was initiated in 1995 and currently covers almost all population (approximately, 23 million people). The research data of Taiwan's National Health Insurance Program constitute a real-world database that has been previously evaluated and the related research articles have been accepted in the journals throughout the world [13, 14, 18–20]. For the protection of personal privacy, patient identification was decoded and scrambled in this study, which was also reviewed and approved by the Institutional Review Board of Taipei Medical University (TMU-JIRB-201905042; TMUJIRB-201902053) and E-DA Hospital (EDA-JIRB-2019001).

### 2.2. Study Design

In this real-world database that included a retrospective cohort of 23 million insured individuals, we identified 47235 patients (aged 20 years and older) who first used RYR prescriptions between 2010 and 2014 and who were without a history of stroke before the date of RYR use and categorized them as the RYR cohort. During the same index period, 50,886 patients used lovastatin (aged 20 years and older) who did not use RYR prescriptions were identified. Both groups were matched by age and sex (with a case-control ratio = 1 : 1), and they had no history of stroke before the index date; thus, there were 34,723 patients with RYR prescriptions and 34,723 patients without RYR prescriptions for comparison. Patients with any physicians' diagnoses (including primary and secondary diagnoses during inpatient and outpatient care) of stroke before the use of RYR prescriptions or the index date were excluded, in order to ensure that all of the study participants were free of stroke at the start of both cohorts. The follow-up started from the time of the use of RYR prescriptions or the index date and lasted until the occurrence of stroke events, censoring due to death, or loss to follow-up by December 31, 2017. Therefore, no immortal time bias existed in this study. We sought to evaluate the risk of incident stroke between the RYR cohort and the comparison cohort during the follow-up period.

### 2.3. Definitions and Criteria

The use of RYR was defined as people who visited clinics of traditional Chinese medicine and who received a physician's prescription for RYR (LipoCol Forte®) under the coverage of Taiwan's National Health Insurance Program. The prescription of RYR is an all-natural RYR extract that has been scientifically processed and the related details were described in the previous studies [13, 14].

The main outcome of incident stroke (430–437) and medical conditions was identified by using physicians' diagnoses and the International Classification of Diseases, Ninth Revision, Clinical Modification (ICD-9-CM). We defined stroke cases as people who had the first occurrence of stroke and who received inpatient care by neurologists and/or neurosurgeons during stroke hospitalization and during the follow-up period. Coexisting medical conditions were determined from medical claims and included hypertension (401–405), mental disorders (290–319), diabetes (250), chronic obstructive pulmonary disease (490, 491, 496), ischemic heart disease (410–414), liver cirrhosis (571.2, 571.5, 571.6), heart failure (428), and renal dialysis (administration codes, D8 and D9). The Charlson comorbidity index, emergency visits, and number of hospitalizations were also considered to be indicators of personal medical conditions in this study. The low income statuses of patients were identified by the definition of the Ministry of Health and Welfare, Taiwan.

### 2.4. Statistical Analysis

We used Chi-square tests to compare the baseline characteristics between people with and without the use of RYR prescriptions. Using multivariate Cox proportional hazard regressions, we conducted different adjusted models of the covariates (age, sex, low income, hypertension, mental disorders, diabetes, chronic obstructive pulmonary disease, ischemic heart disease, liver cirrhosis, heart failure, and renal dialysis) to calculate the corresponding adjusted hazard ratios (HRs) and 95% confidence intervals (CIs) of incident stroke that were associated with the use of RYR prescriptions. The risk of incident stroke between people who did and did not use RYR prescriptions was also calculated in the subgroup analyses by age, sex, number of medical conditions, emergency visits, hospitalizations, and the Charlson comorbidity index. In addition, we performed sensitivity analyses and excluded incident cases of stroke within the initial one-, two-, and three-month follow-up periods.

## 3. Results

The characteristics of people with and without the use of RYR prescriptions are shown in Table 1. Under frequency matching by age and sex, compared to the non-RYR group, the RYR cohort had lower proportions of low income (*p* < 0.0001) and medical conditions, including hypertension (*p* < 0.0001), diabetes (*p* < 0.0001), ischemic heart disease (*p* < 0.0001), chronic obstructive pulmonary disease (*p* < 0.0001), liver cirrhosis (*p* < 0.0001), heart failure (*p* < 0.0001), renal dialysis (*p* < 0.0001), and the Charlson comorbidity index scores ≥3 (*p* < 0.0001). The proportions of hospitalizations ≥3 times (*p* < 0.0001), emergency visits ≥3 (*p* < 0.0001), the use of anti-hypertension drugs (*p* < 0.0001), and the use of anticoagulant drugs (*p* < 0.0001) were lower in people who had RYR than in those individuals without RYR. The unmatched characteristics of people with and without the use of RYR prescriptions are shown in Table S1.

In Table 2, compared with those individuals who did not use RYR, people who used RYR prescriptions had a lower risk of stroke during the follow-up period (HR 0.65, 95% CI 0.59–0.71). The use of RYR prescriptions was also associated with a decreased risk of hemorrhagic stroke (HR 0.60, 95% CI 0.44–0.83), ischemic stroke (HR 0.49, 95% CI 0.43–0.57), and other types of strokes (HR 0.53, 95% CI 0.42–0.67). After excluding incident stroke cases during the initial one month (HR 0.66, 95% CI 0.60–0.73), two months (HR 0.67, 95% CI 0.61–0.74), and three months (HR 0.68, 95% CI 0.61–0.75) of the follow-up period, the use of RYR prescriptions was also associated with a decreased stroke risk.

The stratified analysis (Table 3) revealed that the association between the use of RYR prescriptions and a decreased risk of stroke was significant in women (HR 0.66; 95% CI 0.58–0.77), men (HR 0.63; 95% CI 0.55–0.72), and those people aged more than 20 years. The adjusted HRs of stroke risk that were associated with RYR prescription use among people with 0, 1, 2, and ≥3 medical conditions were 0.39 (95% CI 0.31–0.50), 0.58 (95% CI 0.50–0.68), 0.68 (95% CI 0.57–0.81), and 0.74 (95% CI 0.60–0.92), respectively. The association between the use of RYR prescriptions was associated with a decreased risk of stroke in people with the Charlson comorbidity index scores of 0 (HR 0.56, 95% CI 0.46–0.68), 1 (HR 0.70, 95% CI 0.59–0.84), 2 (HR 0.71, 95% CI 0.56–0.90), and ≥3 (HR 0.66, 95% CI 0.55–0.79). The use of RYR prescriptions was associated with the decreased stroke risk among people with emergency visits and hospitalizations. In Table 4, there was a potential dose-response relationship between the frequencies of RYR use and decreased stroke risk with an HR of 0.57 (95% CI 0.50–0.64).

## 4. Discussion

This was the first study to compare the risk of incident stroke between people who did and did not use RYR prescriptions. We found a decreased risk of incident stroke in people who used RYR prescriptions, with significant findings observed regardless of age, gender, or medical conditions. Decreased risks of hemorrhagic stroke and ischemic stroke associated with the use of RYR prescriptions were also found in this study.

We propose several reasons to explain the reduction in the stroke risk in patients using RYR prescriptions in this study. First, a meta-analysis of randomized trials of statins (including six trials involving lovastatin) demonstrated large reductions in cholesterol and clear evidence of benefits regarding stroke [21]. The herbal medicine RYR contains monacolin K (lovastatin); thus, the mechanism regarding the lowering of the level of total cholesterol in patients who used RYR is clearly obvious [7, 9, 10, 21]. Undoubtedly, the reduction of the high level of total cholesterol to a normal range is one of the key points of reducing carotid atherosclerosis and subsequent stroke risk [22].

Second, in addition to conventional medicine (also known as biochemical medicine or Western medicine), traditional Chinese medicine is the second most common medical choice in Taiwan, Korea, Japan, and China. People with hyperlipidemia who choose second opinions and receive therapy with RYR prescriptions (as prescribed by physicians with specialties in traditional Chinese medicine) may have better knowledge, attitudes, and practices regarding disease prevention and health promotion [23]; therefore, these factors may also contribute to the decreased incidence of stroke.

Third, an animal study suggested that RYR was effective in combatting obesity-related inflammation, insulin resistance, and nonalcoholic fatty liver diseases in mice, irrespective of monacolin K levels [24]. Our previous analyses also provided some potential evidence to support the hypothesis that a decreased risk of incident diabetes was found in people who used RYR prescriptions [14]. It is believed that the reduction of the risk of diabetes is helpful for the prevention of stroke [25]. However, the results of the current study could not be fully explained by the previously described reasons. In addition to monacolin K, RYR prescriptions contain gamma-aminobutyric acid and various monacolins, phytosterols, and isoflavones [7, 26], and very little is known about the effects of these constituents on atherosclerosis or stroke. We suggest that future studies should evaluate the effects of other components of RYR on inflammation, hyperlipidemia, atherosclerosis, and stroke.

Despite the beneficial effects of RYR on hypercholesterol, as well as the fact that some studies have suggested that RYR is relatively safe [7, 11, 12, 15], the potential side effects of RYR should be considered, such as muscle symptoms, central nervous system complaints, and diabetes, with these effects being partially similar to the effects of statins [27]. Some studies and case reports have indicated that dietary supplements containing red yeast rice may be related to hepatotoxicity [28–30], symptomatic myopathy [28, 30, 31], and erectile dysfunction [32]. Therefore, the uncertain safety of the dietary intake of RYR was indicated by the European Food Safety Authority (EFSA) and other studies [9, 10, 33]. However, the side effects of RYR could not be independently investigated because several clinical trials combined RYR use with other dietary supplements [26, 34, 35]. Unlike dietary supplements, it was suggested that the content of RYR prescriptions with good manufacturing practices were relatively stable and safe, such as Xuezhikang®, HypoCol®, and LipoCol Forte® [7, 11, 12]. Thus, we suggest that the continuous surveillance of adverse reactions from RYR use should become a priority of future trials.

In this study, we used statistical methods and procedures to analyze a real-world database for evaluating the risk of incident stroke in patients using RYR prescriptions. To reduce confounding biases, we used four models of multivariate Cox proportional hazard regressions to adjust for sociodemographic factors, medical conditions, the Charlson comorbidity index, and the use of medical care as potential confounders. To avoid immortal time biases, we calculated the person years in the RYR cohort, beginning from the date of medication intake until the end of the study during the follow-up period. To validate new-onset stroke cases, we conducted sensitivity analyses in three models, which excluded stroke cases within the initial one-, two-, and three-month follow-up periods. To test the reliability, we performed stratified analyses to present the use of RYR associated with decreased stroke risk in various subgroups by age, sex, medical conditions, and use of medical care. To demonstrate the potential dose-response relationship, we calculated the cumulative use of RYR prescriptions and the decreased stroke risk.

The main limitation of our study was the unavailable information concerning patients' compliance with RYR use. Thus, we did not understand whether the study subjects took all of their RYR prescriptions. Second, detailed information on socioeconomics, lifestyle habits (such as smoking and drinking of alcohol), physical activity, eating habits, biochemical measures (such as fasting sugar and lipid levels), and the severity of comorbid disease was not available from the insurance database. Third, the incident stroke and coexisting medical conditions were identified according to the physician's diagnosis during patient visits for medical care. Therefore, we could not exclude the small possibility that very few people with very minor strokes did not seek medical care; thus, we could not identify these stroke cases. Fourth, the unavailable data concerning knowledge, attitudes, and practices regarding disease prevention and family care are also limitations. In addition, we could not evaluate whether more than half of the participants were older than 60-years-old and which individuals may need to take more than one medication, as well as the fact that these medications may influence the control of medical condition and stroke occurrence. Another study limitation involves the possibility that we could not consider all medication use of the participants in this study. In addition, our study was an observational retrospective cohort study that could not provide causal inferences or concrete evidence between the use of RYR prescriptions and stroke risk. Finally, healthy worker effects in the RYR cohort and residual confounding biases were also possible in this study.

In conclusion, we indicated the possibility that people who used RYR prescriptions may have a lower risk of incident stroke, compared to non-RYR users, in this observational study. The decreased risk of incident stroke varied within people with levels of cumulative consumption of RYR prescriptions. However, the outcomes after stroke were not associated with the use of RYR prescriptions. Some limitations need to be cautiously considered when interpreting our findings. We suggest that large multicenter trials be conducted to provide further safety assessments and concrete evidence to demonstrate the relationship between RYR prescriptions and the incident risk of stroke.

## Figures and Tables

**Table 1:** Baseline characteristics of people with and without use of RYR prescription.

	No RYR^*∗*^ *N* = 34723		RYR prescription *N* = 34723		*p* value
Sex	*n*	(%)	*n*	(%)	1.0000
Female	19279	(55.5)	19279	(55.5)	
Male	15444	(44.5)	15444	(44.5)	
Age, years					1.0000
20–29	503	(1.5)	503	(1.5)	
30–39	2115	(6.1)	2115	(6.1)	
40–49	6814	(19.6)	6814	(19.6)	
50–59	14476	(41.7)	14476	(41.7)	
60–69	7768	(22.4)	7768	(22.4)	
70–79	2563	(7.4)	2563	(7.4)	
≥80	484	(1.4)	484	(1.4)	
Low income					<0.0001
No	33397	(96.2)	33937	(97.7)	
Yes	1326	(3.8)	786	(2.3)	
Medical conditions					
Hypertension	17302	(49.8)	13568	(39.1)	<0.0001
Mental disorders	10753	(31.0)	10816	(31.2)	0.6054
Diabetes	14608	(42.1)	7296	(21.0)	<0.0001
Heart failure	1187	(3.4)	697	(2.0)	<0.0001
Renal dialysis	700	(2.0)	188	(0.5)	<0.0001
Ischemic heart disease	6347	(18.3)	5338	(15.4)	<0.0001
COPD	5267	(15.2)	4622	(13.3)	<0.0001
Liver cirrhosis	2347	(6.8)	1845	(5.3)	<0.0001
CCI, score					<0.0001
0	6565	(18.9)	10019	(28.9)	
1	9086	(26.2)	8181	(23.6)	
2	6118	(17.6)	6138	(17.7)	
≥3	12954	(37.3)	10385	(29.9)	
Number of hospitalizations					<0.0001
0	19271	(55.5)	23189	(66.8)	
1	6829	(19.7)	6306	(18.2)	
2	3304	(9.5)	2483	(7.2)	
≥3	5319	(15.3)	2745	(7.9)	
Number of emergency visits					<0.0001
0	13454	(38.8)	16545	(47.7)	
1	7718	(22.2)	8140	(23.4)	
2	4514	(13.0)	3974	(11.4)	
≥3	9037	(26.0)	6064	(17.5)	
Use of antihypertensive drug	13603	(39.2)	9328	(26.9)	<0.0001
Use of anticoagulant drug	1415	(4.1)	755	(2.2)	<0.0001

CCI, the Charlson comorbidity index; COPD, chronic obstructive pulmonary disease; RYR, red yeast rice. ^∗^denotes the non-RYR group who used lovastatin.

**Table 2:** Risk of incident stroke between RYR cohort and non-RYR group during the follow-up period.

				Incident stroke			
		*n*	PYs	Events	Incidence^*∗*^	HR	(95% CI)†
All stroke	No RYR	34723	212022	1482	6.99	1.00	(Reference)
	RYR	34723	182415	724	3.97	0.65	(0.59–0.71)
Hemorrhagic stroke	No RYR	34723	215445	267	1.24	1.00	(Reference)
	RYR	34723	183391	160	0.87	0.60	(0.44–0.83)
Ischemic stroke	No RYR	34723	208929	1374	6.58	1.00	(Reference)
	RYR	34723	180867	602	3.33	0.49	(0.43–0.57)
Other stroke	No RYR	34723	214373	421	1.96	1.00	(Reference)
	RYR	34723	182865	245	1.34	0.53	(0.42–0.67)

CI, confidence interval; HR, hazard ratio; PYs, person-years; RYR, red yeast rice. ^*∗*^Per 1000 person-years. †Adjusted for all covariates listed in Table 1. Sensitivity analysis: after excluding the incident stroke cases during initial one month, two months, and three months, the risks of stroke associated RYR were 0.66 (95% CI 0.60–0.73), 0.67 (95% CI 0.61–0.74), and 0.68 (95% CI 0.61–0.75), respectively.

**Table 3:** Stratified analysis for the association between risk of stroke and RYR prescription.

				Incident stroke			
		*n*	PYs	Events	Incidence^*∗*^	HR	(95% CI)†
Women	No RYR	19279	118911	708	5.95	1.00	(Reference)
	RYR	19279	101040	341	3.37	0.66	(0.58–0.77)
Men	No RYR	15444	93111	774	8.31	1.00	(Reference)
	RYR	15444	81375	383	4.71	0.63	(0.55–0.72)
Age, 20–39 years	No RYR	2618	16407	33	2.01	1.00	(Reference)
	RYR	2618	14300	11	0.77	0.44	(0.21–0.93)
Age, 40–49 years	No RYR	6814	42595	196	4.60	1.00	(Reference)
	RYR	6814	36873	58	1.57	0.46	(0.33–0.63)
Age, 50–59 years	No RYR	14476	89266	513	5.75	1.00	(Reference)
	RYR	14476	76679	248	3.23	0.65	(0.55–0.77)
Age, 60–69 years	No RYR	7768	46372	444	9.57	1.00	(Reference)
	RYR	7768	39530	213	5.39	0.64	(0.53–0.76)
Age, ≥70 years	No RYR	3047	17382	296	17.0	1.00	(Reference)
	RYR	3047	15033	194	12.9	0.80	(0.66–0.98)
Medical conditions, 0	No RYR	4817	28186	192	6.81	1.00	(Reference)
	RYR	9844	49777	122	2.45	0.39	(0.31–0.50)
Medical conditions, 1	No RYR	12363	73476	548	7.46	1.00	(Reference)
	RYR	12171	63303	260	4.11	0.58	(0.50–0.68)
Medical conditions, 2	No RYR	9916	61299	428	6.98	1.00	(Reference)
	RYR	7748	41539	208	5.01	0.68	(0.57–0.81)
Medical conditions, ≥3	No RYR	7627	49061	314	6.40	1.00	(Reference)
	RYR	4960	27796	134	4.82	0.74	(0.60–0.92)
CCI score, 0	No RYR	6565	38085	332	8.72	1.00	(reference)
	RYR	10019	50253	195	3.88	0.56	(0.46–0.68)
CCI score, 1	No RYR	9086	54107	435	8.04	1.00	(Reference)
	RYR	8181	42426	205	4.83	0.70	(0.59–0.84)
CCI score, 2	No RYR	6118	37765	251	6.65	1.00	(Reference)
	RYR	6138	32766	131	4.00	0.71	(0.56–0.90)
CCI score, ≥3	No RYR	12954	82065	464	5.65	1.00	(Reference)
	RYR	10385	56970	193	3.39	0.66	(0.55–0.79)
Emergency visit, 0	No RYR	13454	79028	589	7.45	1.00	(Reference)
	RYR	16545	84041	255	3.03	0.48	(0.41–0.56)
Emergency visits, 1	No RYR	7718	47264	317	6.71	1.00	(Reference)
	RYR	8140	43000	207	4.81	0.85	(0.70–1.03)
Emergency visits, ≥2	No RYR	13551	85730	576	6.72	1.00	(Reference)
	RYR	10038	55374	262	4.73	0.75	(0.64–0.88)
Hospitalizations, 0	No RYR	19271	115388	835	7.24	1.00	(Reference)
	RYR	23189	119380	490	4.10	0.69	(0.61–0.78)
Hospitalizations, 1	No RYR	6829	42523	293	6.89	1.00	(Reference)
	RYR	6306	34280	115	3.35	0.57	(0.45–0.72)
Hospitalizations, ≥2	No RYR	8623	54111	354	6.54	1.00	(Reference)
	RYR	5228	28755	119	4.14	0.63	(0.50–0.79)

CCI, the Charlson comorbidity index; CI, confidence interval; HR, hazard ratio; PYs, person years; RYR, red yeast rice. ^*∗*^Per 100 person-years. †Adjusted for all covariates listed in Table 1.

**Table 4:** Risk of stroke between people used frequency of RYR prescription.

			Incident stroke			
	*n*	PYs	Events	Incidence^*∗*^	HR	(95% CI)†
Non-RYR group (used lovastatin)	34723	212022	1482	6.99	1.00	(Reference)
Frequency of RYR use						
1	10184	52023	225	4.33	0.72	(0.62–0.83)
2	4906	25149	112	4.45	0.74	(0.61–0.90)
3	3382	17281	74	4.28	0.73	(0.57–0.92)
≥4	16251	87962	313	3.56	0.57	(0.50–0.64)

CI, confidence interval; HR, hazard ratio; PYs, person years; RYR, red yeast rice. ^*∗*^Per 1000 person-years. †Adjusted for all covariates listed in Table 1; the dose-response relationship (*p* for trend<0.0001).

## Data Availability

The data underlying this study is from the Health and Welfare Data Science Center. Interested researchers can obtain the data through formal application to the Health and Welfare Data Science Center, Department of Statistics, Ministry of Health and Welfare, Taiwan (https://dep.mohw.gov.tw/dos/lp-2506-113.html). Under the regulations from the Health and Welfare Data Science Center, we have made the formal application (included application documents, study proposals, and ethics approval of the institutional review board) of the current insurance data from in 2019. The authors of the present study had no special access privileges in accessing the data which other interested researchers would not have.

## Abbreviations

ICD-9-CM: International Classification of Diseases, 9th revision, clinical modification
CI: Confidence interval
RYR: Red yeast rice
HR: Hazard ratio.

## Supplementary Materials

Supplementary Materials Supplementary Description Table 1 Characteristics of patients with use of RYR prescription and lovastatin before matching by propensity score.
