# Supplementary material for: Decreased Risk of Stroke in People Using Red Yeast Rice Prescriptions (LipoCol Forte®): a Total Population-Based Retrospective Cohort Study
Source: Evid Based Complement Alternat Med. 2022 Apr 23;2022:8160425. doi: 10.1155/2022/8160425 (PMC9056220; doi:10.1155/2022/8160425)
Supplement: Supplementary Materials — Supplementary Description Table 1 Characteristics of patients with use of RYR prescription and lovastatin before matching by propensity score. [file 8160425.f1.doc]

| **TABLE S1** Characteristics of patients with use of RYR prescription and lovastatin before matching by propensity score | | | | | |
| --- | --- | --- | --- | --- | --- |
|  | Lovastatin  N=50886 | | RYR  N=47235 | | p-value |
| Sex | n | (%) | n | (%) | <0.0001 |
| Female | 27978 | (55.0) | 26733 | (56.6) |  |
| Male | 22908 | (45.0) | 20502 | (43.4) |  |
| Age, years |  |  |  |  | <0.0001 |
| 20-29 | 503 | (1.0) | 2300 | (4.9) |  |
| 30-39 | 2115 | (4.2) | 6904 | (14.6) |  |
| 40-49 | 6814 | (13.4) | 11914 | (25.2) |  |
| 50-59 | 15136 | (29.7) | 15302 | (32.4) |  |
| 60-69 | 13349 | (26.2) | 7768 | (16.5) |  |
| 70-79 | 9431 | (18.5) | 2563 | (5.4) |  |
| ≥80 | 3538 | (7.0) | 484 | (1.0) |  |
| Low income |  |  |  |  | <0.0001 |
| No | 49137 | (96.6) | 46005 | (97.4) |  |
| Yes | 1749 | (3.4) | 1230 | (2.6) |  |
| Coexisting medical conditions |  |  |  |  |  |
| Hypertension | 27489 | (54.0) | 16337 | (34.6) | <0.0001 |
| Diabetes | 21763 | (42.8) | 8716 | (18.5) | <0.0001 |
| Mental disorders | 16051 | (31.5) | 14335 | (30.4) | <0.0001 |
| Ischemic heart disease | 10531 | (20.7) | 6157 | (13.0) | <0.0001 |
| COPD | 8914 | (17.5) | 5696 | (12.1) | <0.0001 |
| Liver cirrhosis | 3048 | (6.0) | 2421 | (5.1) | <0.0001 |
| Heart failure | 2529 | (5.0) | 779 | (1.7) | <0.0001 |
| Renal dialysis | 1090 | (2.1) | 221 | (0.5) | <0.0001 |
| Charlson comorbidity index, score |  |  |  |  | <0.0001 |
| 0 | 8680 | (17.1) | 15142 | (32.1) |  |
| 1 | 12699 | (25.0) | 11145 | (23.6) |  |
| 2 | 8713 | (17.1) | 8246 | (17.5) |  |
| ≥3 | 20794 | (40.9) | 12702 | (26.9) |  |
| Number of hospitalizations |  |  |  |  | <0.0001 |
| 0 | 25672 | (50.5) | 32243 | (68.3) |  |
| 1 | 10295 | (20.2) | 8351 | (17.7) |  |
| 2 | 5403 | (10.6) | 3211 | (6.8) |  |
| ≥3 | 9516 | (18.7) | 3430 | (7.3) |  |
| Number of emergency visits |  |  |  |  | <0.0001 |
| 0 | 18264 | (35.9) | 22534 | (47.7) |  |
| 1 | 11126 | (21.9) | 11073 | (23.4) |  |
| 2 | 6771 | (13.3) | 5445 | (11.5) |  |
| ≥3 | 14725 | (28.9) | 8183 | (17.3) |  |
| Anti-hypertension drug use | 21349 | (41.9) | 11676 | (24.7) | <0.0001 |
| Anticoagulant drug use | 2470 | (4.8) | 864 | (1.8) | <0.0001 |
| COPD, chronic obstructive pulmonary disease; RYR, red yeast rice | | | | | |
